# Stakeholder engagement is essential to maximise the impact of research on infant feeding in the context of HIV

**DOI:** 10.1177/20499361211057970

**Published:** 2021-11-29

**Authors:** Bakita Kasadha, Shema Tariq, Farai Nyatsanza, Nell Freeman-Romilly, Angelina Namiba, Tanvi Rai

**Affiliations:** Medical Sociology & Health Experiences Research Group (MS&HERG), Nuffield Department of Primary Care Health Sciences, University of Oxford, Radcliffe Observatory Quarter, Woodstock Road, Oxford OX2 6GG, UK; University College London, London, UK; Cambridgeshire Community Services NHS Trust, Cambridge, UK; Oxford University Hospitals NHS Foundation Trust, Oxford, UK; 4M Network, London, UK; University of Oxford, Oxford, UK

Dear Editor-in-Chief

The 2016 updated World Health Organization (WHO) and UNICEF guidelines on HIV and infant feeding strongly recommend that women and birthing parents living with HIV breastfeed for at least 12 months (and preferably up to 24 months).^1^ These guidelines are primarily intended for lower-income countries with high HIV prevalence, where the potential risk of postpartum transmission of HIV via breast milk is likely to be lower than the risk of infant malnutrition or death as a result of limited access to clean water and/or formula milk. Formula feeding is only considered safe if certain conditions are met including access to clean water, sufficient and reliable supply of formula milk, safe conditions in which to prepare the milk, and family support. In high-income countries, where it is assumed these conditions are met, formula feeding is recommended in the context of HIV.

‘Undetectable equals untransmittable’ or ‘U = U’, is the campaign message highlighting that people living with HIV on antiretroviral therapy (ART) with an undetectable viral load cannot transmit HIV through sex.^2^ However, it is important to note that U = U does *not* currently apply to transmission of HIV through breastfeeding, even when birthing parents are on treatment and virologically suppressed.^3,4^ Maternal ART reduces the risk of infant acquisition of HIV significantly; however, it does not reduce the risk to zero.^5^ PROMISE, a multicentre randomised controlled trial comparing maternal ART with infant nevirapine prophylaxis in sub-Saharan Africa and India, reported two cases of infant HIV acquisition through breastfeeding despite undetectable maternal HIV viral load on ART.^5,6^ In the absence of empirical data in high-income countries, guidance in these countries remains conservative, with formula feeding the preferred choice for birthing parents with HIV in order to avoid all risk of postpartum transmission.

Up until 2012, UK national guidelines advised that a woman living with HIV who wished to breastfeed her baby would constitute grounds for referral to child protection teams;^7^ this is still the case in Canada and parts of the United States,^8,9^ and has prompted North American women’s networks and organisations to advocate for informed infant-feeding decisions.^10^ The most recent 2018 British HIV Association (BHIVA) guidelines on HIV and infant feeding still positions formula feeding as the preferred option, but recognises that some birthing parents may choose to breastfeed, and advise that they are supported to do so when safe.^11^ In practice, however, support for breastfeeding in the context of HIV can be inconsistent; many healthcare professionals (HCPs) still recommend formula feeding, while others may promote informed decision-making but without providing the requisite support or information needed to make such a choice.^12^

We believe that presenting decision-making about infant feeding in the context of HIV as the birthing parent’s ‘choice’ is disingenuous and misleading. By focussing almost exclusively on risks of HIV transmission, infant-feeding guidelines in high-income countries obscure the myriad other factors that shape parents’ choices around infant feeding. BHIVA guidelines only acknowledged the emotional, social and psychological implications of *not* breastfeeding for the first time in 2018.^13^

Parents living with HIV encounter a number of challenges when considering how to feed their babies. These include maintaining discretion about HIV status through maternity and postpartum; managing divergent messaging from HCPs versus family and friends; confusion arising from discrepant local and global guidance; the financial cost of formula feeding; and grief around the loss of breastfeeding experience.^12–16^ All of this is experienced within the context of the unrelenting physical and mental pressures that come with growing, birthing and caring for an infant. In the United Kingdom, over 70% of women living with HIV reported to be pregnant are of Black African ethnicity; they may experience additional intersectional disadvantages as a result of racially minoritised status, such as language barriers, cultural expectations, immigration issues and financial insecurity. Moreover, infant feeding decisions are far from a binary choice made at one point in time; rather they are highly contingent and dynamic, with parents revisiting their decision countless times over the course of new babyhood.^13–16^

Addressing the complex, multidimensional landscape within which infant feeding decisions are located involves new ways of researching. We call for the increased adoption of participatory approaches within HIV research, in order to develop evidence that is practicable and grounded in the reality of people’s lived experiences.

In the case of infant feeding decisions, we are conducting a qualitative study called Nourish-UK (https://www.phc.ox.ac.uk/nourishUK). We aim to explore how women and birthing parents living with HIV decide how to feed their newborn babies in the United Kingdom. Our objectives are to (1) improve understanding of infant feeding decision-making among new mothers and birthing parents living with HIV in the United Kingdom; (2) identify barriers and facilitators to implementing parents’ chosen method of feeding; (3) develop a free, online resource on healthtalk.org for supporting and information targeted at parents living with HIV and their HCPs; and (4) explore acceptability of future research involving the testing of breast milk of new mothers and birthing parents living with HIV (although we will not be testing breast milk in Nourish-UK). Findings will also be used to inform national guidelines.

The study is ongoing; however, we now share our experiences of engaging and working with multiple stakeholders from different sectors, in order to develop a robust study with maximum impact.

Our study team includes academics, clinicians and people with lived experience of HIV and advocacy. We have also established an advisory panel and a Patient and Public Involvement (PPI) panel. The advisory panel comprises over 20 professionals, including key UK HIV clinicians, obstetricians, specialist midwives, HIV charities, policy and support organisations, lactation specialists, doulas and mothers living with HIV. The advisory panel supports recruitment and sampling and provides oversight of the study conduct. The PPI panel is part of the advisory panel and is formed of five mothers living with HIV; some work within the HIV field as Mentor Mothers (supporting mothers throughout pregnancy and postpartum) or as advocates.

Since the study commenced, we have held an advisory panel meeting and a PPI meeting. Our panel members have highlighted the importance of situating conversations about infant feeding and HIV within a historical and international context. For example, PPI panel members recalled how evolving feeding guidelines and data had shaped their own experiences and decisions as mothers living with HIV. They also shared their experiences of inconsistencies in advice and support around infant feeding and HIV from HCPs outside the HIV field and lack of information on the full range of feeding options available to them (i.e. human milk donations), emphasising the importance of effective dissemination of both study findings and guidelines. Furthermore, our panel of experts by experience have foregrounded the importance of using stigma-free language when engaging parents living with HIV.^17,18^

Meanwhile, the HIV specialist HCPs on our team and in the advisory panel have provided important contextual information on existing referral pathways from HIV clinics to peer support, foodbanks and other services. Reflecting on the practice of their peers, they spoke of varying levels of confidence among colleagues when discussing HIV and breastfeeding, partly resulting from limited awareness of latest data and national guidelines.

The midwives and doulas in our advisory panel have identified the lack of consistent and appropriate lactation support for parents living with HIV as a key barrier to supporting parents’ choice in feeding. They also drew our attention to the potential emotional impact of additional surveillance and monitoring for those who choose to breastfeed.

And finally, through our communication with various HIV clinics and organisations that supply formula, we have learned that the well-documented inconsistencies in provision of free formula milk for parents with HIV remain the case across the country,^19^ causing ongoing and additional financial burden and stress for parents.

Our unfolding conversations with stakeholders have allowed us to begin to develop a nuanced understanding of the landscape in which HIV and infant-feeding decisions are being made in the United Kingdom. In response, we have refined our research methods; for example, we have decided to include a small set of new fathers in our interviews in order to understand how decisions are made as a dyad. By engaging with multidisciplinary experts, HIV advocates and experts by experience, we are better positioned to deliver high-quality, impactful research that is grounded in parents’ day-to-day reality. We hope that the knowledge we generate will contribute to improved support and choice for birthing parents living with HIV as they navigate the complexities of infant feeding.

We encourage all study teams to acknowledge the breadth of knowledge that exists beyond clinical and academic spaces, and to engage meaningfully with a wide range of stakeholders at all points of the research process in order to deliver findings that improve the health and well-being of women and birthing parents living with HIV.

## References

[1] WHO, UNICEF. Updates on HIV and infant feeding guideline, 2016, https://www.unicef.org/esa/reports/updates-hiv-and-infant-feeding-guideline

[2] UNAIDS. Undetectable = untransmittable, 2018, https://www.unaids.org/en/resources/presscentre/featurestories/2018/july/undetectable-untransmittable

[3] Waitt C Low N Van de Perre P , et al. Does U=U for breastfeeding mothers and infants? breastfeeding by mothers on effective treatment for HIV infection in high-income settings. Lancet 2018; 5: e531–e536. 10.1016/S2352-3018(18)30098-5 29960731

[4] Freeman-Romilly N Nyatsanza F Namiba A , et al. Moving closer to what women want? a review of breastfeeding and women living with HIV in the UK and high-income countries. HIV Med 2019; 21: 1–8. 10.1111/hiv.12792 31825556

[5] Flynn PM Taha TE Cababasay M , et al. Prevention of HIV-1 transmission through breastfeeding: efficacy and safety of maternal antiretroviral therapy versus infant nevirapine prophylaxis for duration of breastfeeding in HIV-1-infected women with high CD4 cell count (IMPAACT PROMISE): a randomized, open-label, clinical trial. J Acquir Immune Defic Syndr 2018; 77: 383–392. 29239901 10.1097/QAI.0000000000001612 PMC5825265

[6] Flynn PM Taha TE Cababasay M , et al. Association of maternal viral load and CD4 count with perinatal HIV-1 transmission risk during breastfeeding in the PROMISE postpartum component. J Acquir Immune Defic Syndr 2021; 88: 206–213. 34108383 10.1097/QAI.0000000000002744 PMC8434954

[7] Taylor G Clayden P Dhar J , et al. British HIV Association guidelines for the management of HIV infection in pregnant women 2012. HIV Med 2012; 13: 87–157. 22830373 10.1111/j.1468-1293.2012.01030_2.x

[8] Greene S Ion A Kwaramba G , et al. Surviving surveillance: how pregnant women and mothers living with HIV respond to medical and social surveillance. Qual Health Res 2017; 27: 2088–2099. 28814159 10.1177/1049732317725219

[9] Rodríguez-Colón JM . The Madonna’s breast and the surprise of the real: from ideal to embodied. In: Drew P Edwards R (eds) Breasts across motherhood: lived experiences and critical examinations. Toronto, ON, Canada: Demeter Press, 2020, pp. 43–52.

[10] The Well Project, ICW North America. Expert consensus statement calls to advance efforts around infant feeding among women and other birthing parents living with HIV in the United States and Canada. The Well Project, 2020, https://www.thewellproject.org/news-press/expert-consensus-statement-calls-advance-efforts-around-infant-feeding-among-women-and

[11] BHIVA. BHIVA guidelines for the management of HIV in pregnancy and postpartum 2018 (2020 third interim update). BHIVA, 2018, https://www.bhiva.org/pregnancy-guidelines 10.1111/hiv.12720 30869192

[12] 4M Mentor Mothers Network, Salamander Trust. Infant feeding for women living with HIV. 4M Network, 2020, https://4mmm.org/wp-content/uploads/2020/12/4M_Breastfeeding_PaperAugust2020.pdf

[13] Tariq S Elford J Tookey P , et al. ‘It pains me because as a woman you have to breastfeed your baby’: decision-making about infant feeding among African women living with HIV in the UK. Sex Transm Infect 2016; 92: 331–336. 26757986 10.1136/sextrans-2015-052224 PMC4975819

[14] Greene S Ion A Elston D , et al. ‘Why aren’t you breastfeeding?’ how mothers living with HIV talk about infant feeding in a ‘breast is best’ world. Health Care Women Int 2014; 36: 883–901. 24527767 10.1080/07399332.2014.888720

[15] Griswold MK Pagano-Therrien J . Women living with HIV in high income countries and the deeper meaning of breastfeeding avoidance: a metasynthesis. J Human Lact 2020; 36: 44–52. 31895603 10.1177/0890334419886565

[16] Nyatsanza F Gubbin J Gubbin T , et al. Over a third of childbearing women with HIV would like to breastfeed: a UK survey of women living with HIV. Int J STD AIDS 2021; 32: 856–860. 33629917 10.1177/0956462421999951

[17] Dilmitis S Edwards O Hull B , et al. Language, identity and HIV: why do we keep talking about the responsible and responsive use of language? language matters. J Int AIDS Soc 2012; 15(Suppl. 2): 17990.

[18] Watson S Namiba A Lynn V . The language of HIV: a guide for nurses, 2019, https://www.nhivna.org/file/5dcbdcb83254e/BP-19-2.pdf

[19] National AIDS Trust. Policy briefing: access to formula milk for mothers living with HIV in the UK, 2017, https://www.nat.org.uk/sites/default/files/publications/Access%20to%20Formula%20Milk%20Briefing%20FINAL.pdf

